# Bis{2-[bis­(2-hy­droxy­eth­yl)amino]­acetato}­zinc(II) monohydrate

**DOI:** 10.1107/S241431462501003X

**Published:** 2025-11-21

**Authors:** Erik Rakovský, Lenin Thulluvan Valappil, Yogeswara Rao Pateda, Lenka Bartošová, Jana Chrappová

**Affiliations:** a Comenius University in Bratislava, Faculty of Natural Sciences, Department of Inorganic Chemistry, Ilkovičova 6, 84215 Bratislava, Slovak Republic; b Department of Food Databases, Food Research Institute, National Agricultural and Food Centre, Priemyselná 4, 824 75 Bratislava, Slovak Republic; Goethe-Universität Frankfurt, Germany

**Keywords:** crystal structure, bicine, zinc(II), 2-(bis­(2-hy­droxy­eth­yl)amino)­acetate, *N*,*N*-bis­(2-hy­droxy­eth­yl)glycine

## Abstract

The title compound, [Zn(C_6_H_12_NO_4_)_2_]·H_2_O, crystallizes in the monoclinic space group *P*2_1_/*c* with one formula unit per asymmetric unit. The Zn^II^ atom is coordinated by a tertiary amine N atom, a carboxyl­ate O atom and a hy­droxy O atom from each of two bicine ligands, forming a distorted octa­hedral coordination environment. Inter­molecular inter­actions consist of O—H⋯O and weak C—H⋯O hydrogen bonds forming a three-dimensional supra­molecular network.

## Abstract 2

The Zn^II^ atom in the title compound has distorted octa­hedral N_2_O_4_ coordination geometry. The inter­molecular inter­actions in the crystal structure consist of O–H⋯O and weak C–H⋯O hydrogen bonds.

## Graphical abstract

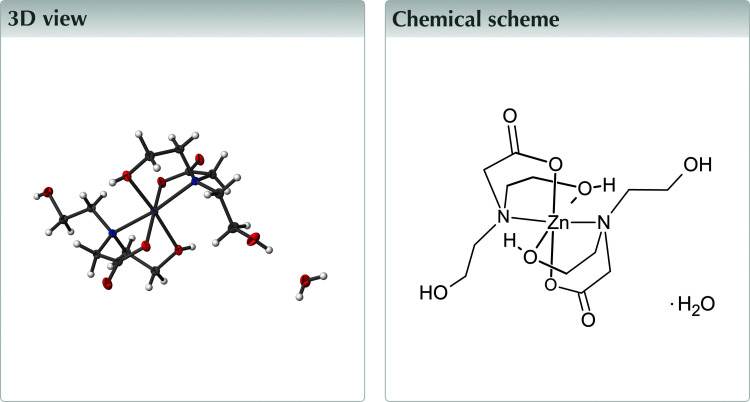

## Structure description

**Figure 1:**
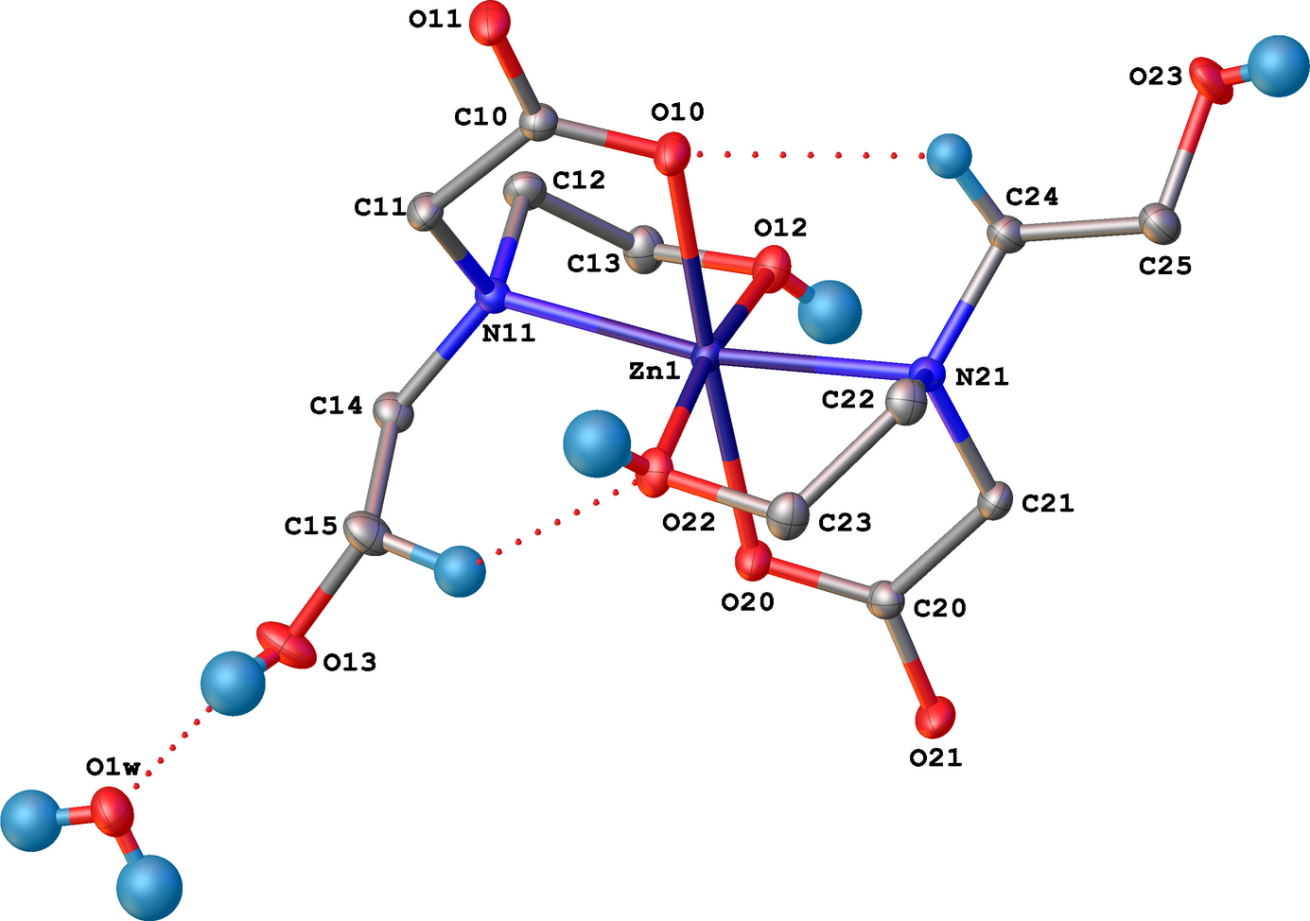
Displacement ellipsoid plot (50% probability) of the asymmetric unit of the title compound with the numbering scheme. Intra­molecular C—H⋯O bonds and the O—H⋯OH_2_ bond are displayed as dashed lines. H atoms belonging to C parent atoms that are not involved in intra­molecular hydrogen bonds are excluded for clarity.

The asymmetric unit of the title complex (Fig. 1 ▸) contains one mol­ecule of [Zn{N(CH_2_CH_2_OH)(CH_2_COO)}_2_] and one water mol­ecule. The Zn^II^ atom is coordinated by a tertiary amine N atom, a carboxyl­ate O atom and one hy­droxy O atom from each of two 2-[bis­(2-hy­droxy­eth­yl)amino]­acetato [*N*,*N*-bis­(2-hy­droxy­eth­yl)glycine, bicine] ligands, thus forming a distorted octa­hedral environment with mean Zn–ligand distance <*D*> = 2.128 Å, polyhedral volume *V* = 12.41 Å and distortion parameters *ζ* = 0.16 Å (distance distortion), *Σ* = 89° (angle distortion), *Θ* = 255° (torsional distortion) and *Δ* = 0.0003 (tilting distortion) (Ketkaew *et al.*, 2021 ▸; Buron-Le Cointe *et al.*, 2012 ▸; Alonso *et al.*, 2000 ▸; McCusker *et al.*, 1996 ▸; Marchivie *et al.*, 2005 ▸). Individual bond lengths are in the range *d*(Zn—O) = 2.0544 (11)–2.1529 (13) Å and *d*(Zn—N) = 2.1478 (13)–2.1484 (14) Å.

**Figure 2:**
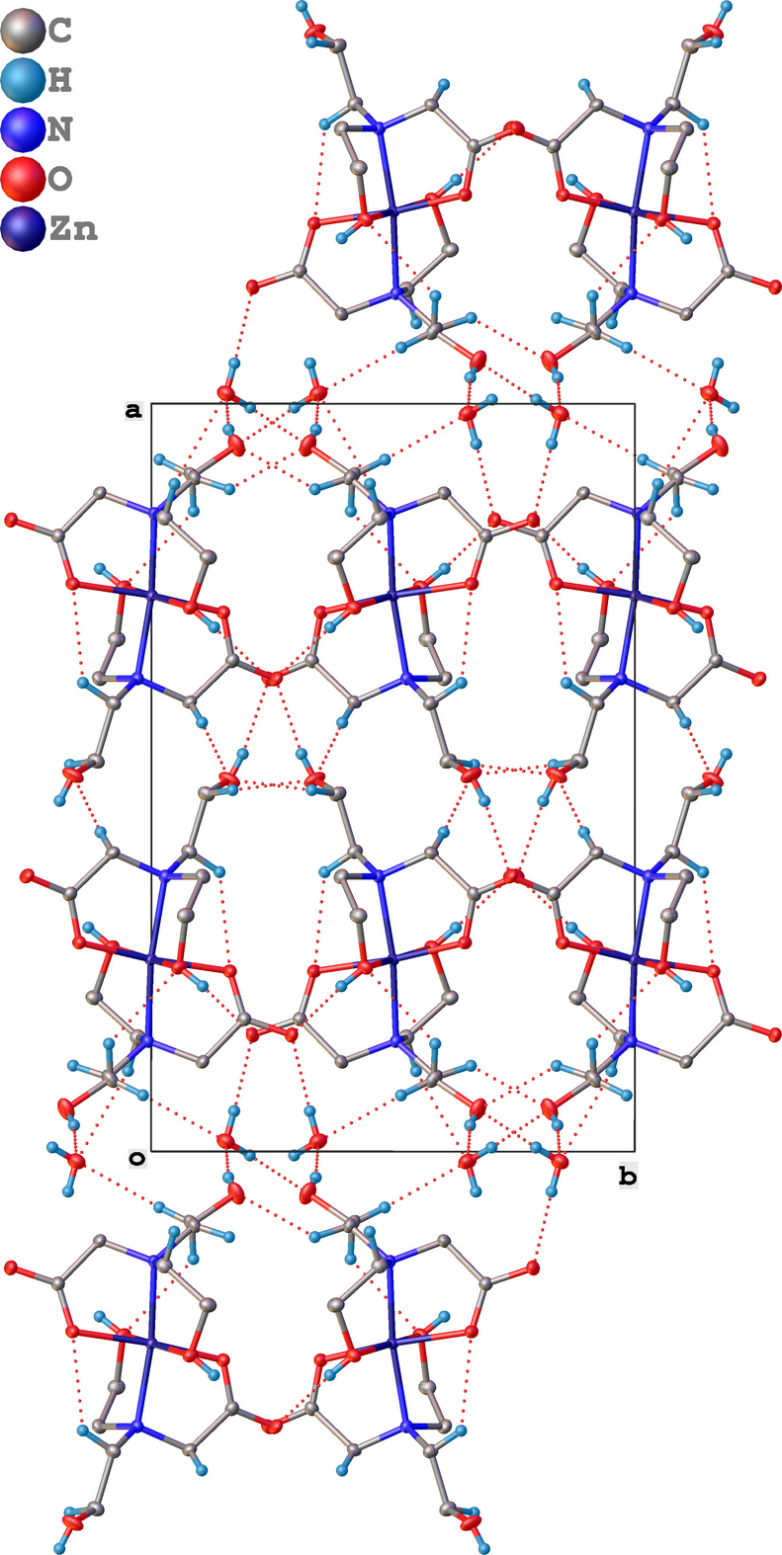
Packing diagram of the title compound along [001] with hydrogen bonds displayed as dashed lines. All non-H atoms are displayed as 50% probability ellipsoids and H atoms as spheres with arbitrary radius. All H atoms not involved in the hydrogen-bonding network are excluded for clarity.

Inter­molecular inter­actions in the structure (Fig. 2 ▸ and Table 1 ▸) consist mainly of medium strength O—H⋯O and weak C—H⋯O hydrogen bonds forming a three-dimensional supra­molecular network (Jeffrey, 1997 ▸). The intra­molecular C—H⋯O hydrogen bonds form two supra­molecular rings, O10–Zn1–N21–C24–H24*b*⋯O10 with graph set N_1_ = *S*(5) and O22–Zn1–N11–C14–C15–H15*a*⋯O22 with graph set N_1_ = *S*(6) (Etter *et al.*, 1990 ▸).

## Synthesis and crystallization

Zinc sulfate hepta­hydrate (ZnSO_4_·7H_2_O, 1.4378 g, 5 mmol) was dissolved in a bicine solution (10 mmol) prepared by dissolving bicine (1.6317 g) in 20 ml of deionized water under magnetic stirring. A hot aqueous solution of ammonium metavanadate (NH_4_VO_3_, 1.1701 g, 10 mmol in 40 ml of water) was then added dropwise to the above reaction mixture under continuous stirring. The resulting reaction mixture was stirred for an additional 30 min. and then filtered to remove any insoluble residues. The pH of the clear filtrate was measured to be 4.8. To the filtrate, 9 ml of ethanol was added, and the solution was left for slow crystallization at 4 °C. Transparent crystals suitable for analysis were obtained after standing for 22 days in the refrigerator.

## Refinement

Crystal data, data collection, and structure refinement details are summarized in Table 2 ▸.

## Supplementary Material

Crystal structure: contains datablock(s) I. DOI: 10.1107/S241431462501003X/bt4186sup1.cif

Structure factors: contains datablock(s) I. DOI: 10.1107/S241431462501003X/bt4186Isup2.hkl

CCDC reference: 2502108

Additional supporting information:  crystallographic information; 3D view; checkCIF report

## Figures and Tables

**Table 1:** Hydrogen-bond geometry (Å, °)

*D*—H⋯*A*	*D*—H	H⋯*A*	*D*⋯*A*	*D*—H⋯*A*
O12—H12⋯O21^i^	0.77 (2)	1.89 (2)	2.6558 (17)	176 (2)
O13—H13⋯O1*w*	0.79 (2)	1.89 (2)	2.6658 (19)	167 (1)
O22—H22⋯O11^ii^	0.78 (2)	1.82 (2)	2.5944 (17)	174 (3)
O23—H23⋯O21^iii^	0.73 (2)	1.99 (2)	2.7166 (17)	171 (3)
O1*w*—H1*w*⋯O13^iv^	0.79 (2)	1.95 (2)	2.739 (2)	177 (2)
O1*w*—H2*w*⋯O11^v^	0.79 (2)	1.97 (2)	2.7577 (18)	174 (2)
C12—H12*a*⋯O1*w*^vi^	0.94 (1)	2.65 (1)	3.563 (2)	163 (1)
C14—H14*b*⋯O13^i^	0.95 (1)	2.63 (1)	3.366 (2)	135 (1)
C15—H15*a*⋯O22	0.96 (1)	2.61 (1)	3.330 (2)	133 (1)
C15—H15*b*⋯O1*w*^vii^	0.96 (1)	2.61 (1)	3.564 (2)	172 (1)
C21—H21*a*⋯O23^viii^	0.94 (1)	2.52 (1)	3.457 (2)	177 (1)
C24—H24*b*⋯O10	0.95 (1)	2.53 (1)	3.171 (2)	125 (1)
C25—H25*a*⋯O23^ii^	0.98 (1)	2.62 (1)	3.589 (2)	170 (1)

Symmetry codes: (i) 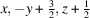; (ii) 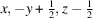; (iii) 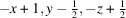; (iv) 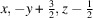; (v) 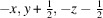; (vi) 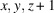; (vii) 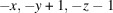; (viii) 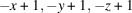.

**Table 2:** Experimental details

Crystal data	
Chemical formula	[Zn(C_6_H_12_NO_4_)_2_]·H_2_O
*M*_r_	407.74
Crystal system, space group	Monoclinic, *P*2_1_/*c*
Temperature (K)	100
*a*, *b*, *c* (Å)	19.0518 (11), 12.1167 (7), 7.1255 (5)
β (°)	99.961 (5)
*V* (Å^3^)	1620.09 (18)
*Z*	4
Radiation type	Mo *K*α
μ (mm^−1^)	1.57
Crystal size (mm)	0.5 × 0.22 × 0.17

Data collection	
Diffractometer	Enraf–Nonius KappaCCD
Absorption correction	Numerical [analytical numeric absorption correction with *PLATON* (Spek, 2020 ▸), using a multifaceted crystal model (de Meulenaer & Tompa, 1965 ▸)]
*T*_min_, *T*_max_	0.462, 0.765
No. of measured, independent and observed [*I* ≥ 2u(*I*)] reflections	16916, 3710, 3003
*R*_int_	0.026
(sin θ/λ)_max_ (Å^−1^)	0.650

Refinement	
*R*[*F*^2^ > 2σ(*F*^2^)], *wR*(*F*^2^), *S*	0.025, 0.056, 1.05
No. of reflections	3710
No. of parameters	248
No. of restraints	6
H-atom treatment	H atoms treated by a mixture of independent and constrained refinement
Δρ_max_, Δρ_min_ (e Å^−3^)	0.44, −0.41

Computer programs: *COLLECT* (Hooft, 1998 ▸), *DIRAX/LSQ* (Duisenberg, 1992 ▸), *EVALCCD* (Duisenberg *et al.*, 2003 ▸), *OLEX2* (Dolomanov *et al.*, 2009 ▸), *OLEX2.refine* (Bourhis *et al.*, 2015 ▸) and *publCIF* (Westrip, 2010 ▸).

## full crystallographic data

### Bis{2-[bis(2-hydroxyethyl)amino]acetato}zinc(II) monohydrate Crystal data

**Table:**

[Zn(C_6_H_12_NO_4_)_2_]·H_2_O	*F*(000) = 858.023
*M**_r_* = 407.74	*D*_x_ = 1.672 Mg m^−^^3^
Monoclinic, *P*2_1_/*c*	Mo *K*α radiation, λ = 0.71073 Å
*a* = 19.0518 (11) Å	Cell parameters from 16916 reflections
*b* = 12.1167 (7) Å	θ = 2.0–27.5°
*c* = 7.1255 (5) Å	µ = 1.57 mm^−^^1^
β = 99.961 (5)°	*T* = 100 K
*V* = 1620.09 (18) Å^3^	Plate, clear colourless
*Z* = 4	0.5 × 0.22 × 0.17 mm

### Bis{2-[bis(2-hydroxyethyl)amino]acetato}zinc(II) monohydrate Data collection

**Table:**

Enraf–Nonius KappaCCD diffractometer	3710 independent reflections
Horizonally mounted graphite crystal monochromator	3003 reflections with *I*≥ 2u(*I*)
Detector resolution: 9 pixels mm^-1^	*R*_int_ = 0.026
ω and θ scans	θ_max_ = 27.5°, θ_min_ = 2.0°
Absorption correction: numerical [analytical numeric absorption correction with PLATON (Spek, 2020), using a multifaceted crystal model (de Meulenaer & Tompa, 1965)]	*h* = −24→24
*T*_min_ = 0.462, *T*_max_ = 0.765	*k* = −15→15
16916 measured reflections	*l* = −9→7

### Bis{2-[bis(2-hydroxyethyl)amino]acetato}zinc(II) monohydrate Refinement

**Table:**

Refinement on *F*^2^	Primary atom site location: iterative
Least-squares matrix: full	Secondary atom site location: difference Fourier map
*R*[*F*^2^ > 2σ(*F*^2^)] = 0.025	Hydrogen site location: difference Fourier map
*wR*(*F*^2^) = 0.056	H atoms treated by a mixture of independent and constrained refinement
*S* = 1.05	*w* = 1/[σ^2^(*F*_o_^2^) + (0.0195*P*)^2^ + 1.1828*P*] where *P* = (*F*_o_^2^ + 2*F*_c_^2^)/3
3710 reflections	(Δ/σ)_max_ = 0.001
248 parameters	Δρ_max_ = 0.44 e Å^−^^3^
6 restraints	Δρ_min_ = −0.41 e Å^−^^3^
34 constraints	

### Bis{2-[bis(2-hydroxyethyl)amino]acetato}zinc(II) monohydrate Special details

**Table:**

Refinement. All non-H atoms were refined anisotropically as a free atoms. H atoms were located in a difference map and refined as riding on their parent atoms with *X*–H distances free to refine and *U*_iso_(H) = 1.2*U*_eq_(C) or 1.5*U*_eq_(O), except of H12 and H22 atoms, which were refined with O12–H12 and O22–H22 distances restrained to be similar and free *U*_iso_ values. Distance similarity restraint was also applied to O13–H13 and O23–H23 rotating groups and O1w–H1w and O1w–H2w distances in the water molecule. All *X*–H distances and *U*_iso_ values refined to physically meaningful values (Cooper, Thompson & Watkin, 2010).

### Bis{2-[bis(2-hydroxyethyl)amino]acetato}zinc(II) monohydrate Fractional atomic coordinates and isotropic or equivalent isotropic displacement parameters (Å^2^)

**Table:**

	*x*	*y*	*z*	*U*_iso_*/*U*_eq_	
Zn1	0.256662 (9)	0.500555 (15)	0.11656 (3)	0.01172 (7)	
N11	0.14734 (7)	0.50656 (11)	0.15470 (18)	0.0116 (3)	
C11	0.11978 (9)	0.39355 (13)	0.1142 (3)	0.0146 (3)	
H11a	0.0808 (6)	0.3813 (2)	0.1788 (9)	0.0175 (4)*	
H11b	0.1028 (3)	0.38609 (17)	−0.0187 (19)	0.0175 (4)*	
C10	0.17703 (9)	0.30740 (13)	0.1770 (2)	0.0140 (3)	
O10	0.24098 (6)	0.33738 (9)	0.20821 (18)	0.0173 (3)	
O11	0.15578 (6)	0.20979 (9)	0.18873 (18)	0.0195 (3)	
C12	0.15266 (9)	0.52755 (14)	0.3639 (2)	0.0155 (4)	
H12a	0.1075 (7)	0.5479 (3)	0.3899 (5)	0.0186 (4)*	
H12b	0.1669 (2)	0.4621 (9)	0.4320 (10)	0.0186 (4)*	
C13	0.20548 (9)	0.61772 (15)	0.4301 (3)	0.0174 (4)	
H13a	0.1909 (2)	0.6866 (10)	0.3631 (9)	0.0209 (4)*	
H13b	0.20878 (10)	0.6302 (2)	0.5669 (19)	0.0209 (4)*	
O12	0.27271 (7)	0.58155 (10)	0.38839 (17)	0.0166 (3)	
H12	0.2992 (12)	0.6301 (18)	0.404 (3)	0.036 (7)*	
C14	0.09927 (9)	0.58948 (13)	0.0470 (2)	0.0150 (3)	
H14a	0.0523 (7)	0.57874 (19)	0.0732 (4)	0.0180 (4)*	
H14b	0.1146 (2)	0.6614 (10)	0.0909 (7)	0.0180 (4)*	
C15	0.09678 (10)	0.58423 (14)	−0.1654 (2)	0.0197 (4)	
H15a	0.1439 (7)	0.58742 (15)	−0.1943 (5)	0.0236 (5)*	
H15b	0.0750 (3)	0.5168 (10)	−0.2154 (8)	0.0236 (5)*	
O13	0.05622 (7)	0.67601 (10)	−0.24655 (19)	0.0247 (3)	
H13	0.0349 (12)	0.6596 (8)	−0.349 (3)	0.0370 (5)*	
N21	0.36772 (7)	0.47131 (11)	0.11406 (19)	0.0122 (3)	
C21	0.39907 (9)	0.57966 (13)	0.0786 (2)	0.0142 (3)	
H21a	0.4267 (4)	0.6049 (4)	0.1931 (16)	0.0171 (4)*	
H21b	0.4299 (5)	0.57005 (19)	−0.0103 (13)	0.0171 (4)*	
C20	0.34408 (9)	0.66775 (13)	0.0038 (2)	0.0140 (3)	
O20	0.27921 (6)	0.64901 (9)	0.00100 (17)	0.0160 (3)	
O21	0.36816 (6)	0.75698 (10)	−0.04897 (18)	0.0200 (3)	
C22	0.36783 (9)	0.39292 (14)	−0.0460 (2)	0.0158 (3)	
H22a	0.4150 (7)	0.38925 (15)	−0.0773 (5)	0.0190 (4)*	
H22b	0.35534 (19)	0.3203 (10)	−0.0076 (6)	0.0190 (4)*	
C23	0.31539 (9)	0.42852 (15)	−0.2202 (2)	0.0175 (4)	
H23a	0.3310 (2)	0.4976 (10)	−0.2683 (7)	0.0210 (4)*	
H23b	0.31361 (9)	0.3734 (8)	−0.3195 (14)	0.0210 (4)*	
O22	0.24593 (6)	0.44215 (10)	−0.17235 (18)	0.0167 (3)	
H22	0.2209 (13)	0.3934 (19)	−0.211 (4)	0.040 (7)*	
C24	0.40087 (9)	0.42152 (14)	0.2986 (2)	0.0151 (3)	
H24a	0.39788 (10)	0.4736 (7)	0.3972 (14)	0.0181 (4)*	
H24b	0.3734 (4)	0.3587 (9)	0.3214 (4)	0.0181 (4)*	
C25	0.47799 (9)	0.38602 (14)	0.3146 (2)	0.0174 (4)	
H25a	0.48311 (12)	0.3319 (8)	0.2150 (14)	0.0209 (4)*	
H25b	0.5085 (4)	0.4499 (9)	0.3011 (3)	0.0209 (4)*	
O23	0.49697 (7)	0.33820 (11)	0.49799 (18)	0.0226 (3)	
H23	0.5314 (11)	0.3106 (19)	0.5051 (12)	0.0339 (4)*	
O1w	−0.01344 (8)	0.65441 (11)	−0.6028 (2)	0.0224 (3)	
H1w	0.0053 (13)	0.7042 (19)	−0.647 (3)	0.039 (7)*	
H2w	−0.0538 (12)	0.6717 (19)	−0.619 (3)	0.034 (7)*	

### Bis{2-[bis(2-hydroxyethyl)amino]acetato}zinc(II) monohydrate Atomic displacement parameters (Å^2^)

**Table:**

	*U*^11^	*U*^22^	*U*^33^	*U*^12^	*U*^13^	*U*^23^
Zn1	0.00975 (10)	0.01046 (10)	0.01519 (10)	−0.00058 (7)	0.00284 (7)	0.00073 (8)
N11	0.0119 (6)	0.0107 (6)	0.0121 (6)	0.0008 (6)	0.0016 (5)	0.0003 (5)
C11	0.0123 (8)	0.0124 (8)	0.0186 (9)	−0.0016 (6)	0.0018 (7)	0.0020 (7)
C10	0.0137 (8)	0.0130 (8)	0.0154 (8)	0.0001 (6)	0.0026 (7)	0.0014 (6)
O10	0.0120 (6)	0.0134 (6)	0.0263 (7)	0.0003 (5)	0.0026 (5)	0.0037 (5)
O11	0.0153 (6)	0.0120 (6)	0.0301 (7)	−0.0010 (5)	0.0008 (5)	0.0045 (5)
C12	0.0165 (8)	0.0178 (8)	0.0127 (8)	0.0008 (7)	0.0043 (7)	0.0007 (6)
C13	0.0151 (8)	0.0203 (9)	0.0170 (9)	0.0013 (7)	0.0033 (7)	−0.0026 (7)
O12	0.0136 (6)	0.0165 (6)	0.0196 (6)	−0.0023 (5)	0.0026 (5)	−0.0030 (5)
C14	0.0146 (8)	0.0121 (8)	0.0180 (9)	0.0024 (7)	0.0021 (7)	0.0011 (7)
C15	0.0212 (9)	0.0184 (9)	0.0191 (9)	0.0076 (7)	0.0026 (7)	0.0029 (7)
O13	0.0333 (8)	0.0196 (6)	0.0184 (7)	0.0087 (6)	−0.0033 (6)	0.0036 (5)
N21	0.0122 (7)	0.0112 (6)	0.0132 (7)	0.0002 (5)	0.0021 (5)	0.0011 (5)
C21	0.0113 (8)	0.0146 (8)	0.0166 (8)	−0.0021 (6)	0.0018 (7)	0.0017 (7)
C20	0.0145 (8)	0.0138 (8)	0.0129 (8)	−0.0016 (6)	0.0003 (7)	0.0001 (6)
O20	0.0123 (6)	0.0128 (5)	0.0226 (6)	0.0000 (5)	0.0019 (5)	0.0033 (5)
O21	0.0147 (6)	0.0149 (6)	0.0295 (7)	−0.0029 (5)	0.0013 (5)	0.0072 (5)
C22	0.0149 (8)	0.0149 (8)	0.0180 (9)	0.0020 (7)	0.0041 (7)	−0.0030 (7)
C23	0.0163 (8)	0.0208 (9)	0.0159 (8)	−0.0007 (7)	0.0040 (7)	−0.0034 (7)
O22	0.0137 (6)	0.0170 (6)	0.0185 (6)	−0.0028 (5)	0.0006 (5)	−0.0045 (5)
C24	0.0140 (8)	0.0163 (8)	0.0149 (8)	0.0017 (7)	0.0023 (7)	0.0031 (7)
C25	0.0157 (8)	0.0196 (8)	0.0164 (8)	0.0031 (7)	0.0015 (7)	0.0033 (7)
O23	0.0163 (6)	0.0306 (7)	0.0201 (6)	0.0102 (6)	0.0008 (5)	0.0077 (6)
O1w	0.0167 (7)	0.0220 (7)	0.0272 (8)	0.0030 (6)	0.0004 (6)	0.0041 (6)

### Bis{2-[bis(2-hydroxyethyl)amino]acetato}zinc(II) monohydrate Geometric parameters (Å, º)

**Table:**

Zn1—N11	2.1478 (13)	C15—O13	1.419 (2)
Zn1—O10	2.1195 (11)	O13—H13	0.79 (2)
Zn1—O12	2.1455 (12)	N21—C21	1.482 (2)
Zn1—N21	2.1484 (14)	N21—C22	1.485 (2)
Zn1—O20	2.0544 (11)	N21—C24	1.485 (2)
Zn1—O22	2.1529 (13)	C21—H21a	0.942 (13)
N11—C11	1.477 (2)	C21—H21b	0.942 (13)
N11—C12	1.499 (2)	C21—C20	1.526 (2)
N11—C14	1.481 (2)	C20—O20	1.253 (2)
C11—H11a	0.950 (13)	C20—O21	1.257 (2)
C11—H11b	0.950 (13)	C22—H22a	0.964 (14)
C11—C10	1.520 (2)	C22—H22b	0.964 (14)
C10—O10	1.254 (2)	C22—C23	1.515 (2)
C10—O11	1.258 (2)	C23—H23a	0.970 (13)
C12—H12a	0.944 (13)	C23—H23b	0.970 (13)
C12—H12b	0.944 (13)	C23—O22	1.432 (2)
C12—C13	1.505 (2)	O22—H22	0.78 (2)
C13—H13a	0.977 (14)	C24—H24a	0.953 (13)
C13—H13b	0.977 (14)	C24—H24b	0.953 (13)
C13—O12	1.433 (2)	C24—C25	1.516 (2)
O12—H12	0.77 (2)	C25—H25a	0.983 (14)
C14—H14a	0.954 (13)	C25—H25b	0.983 (14)
C14—H14b	0.954 (13)	C25—O23	1.418 (2)
C14—C15	1.507 (2)	O23—H23	0.73 (2)
C15—H15a	0.957 (14)	O1w—H1w	0.79 (2)
C15—H15b	0.957 (14)	O1w—H2w	0.79 (2)
			
O10—Zn1—N11	78.53 (5)	H15a—C15—C14	110.27 (10)
O12—Zn1—N11	81.96 (5)	H15b—C15—C14	110.27 (10)
O12—Zn1—O10	98.87 (5)	H15b—C15—H15a	108.5
N21—Zn1—N11	169.94 (5)	O13—C15—C14	107.24 (14)
N21—Zn1—O10	92.31 (5)	O13—C15—H15a	110.27 (10)
N21—Zn1—O12	95.52 (5)	O13—C15—H15b	110.27 (9)
O20—Zn1—N11	107.20 (5)	H13—O13—C15	109.5
O20—Zn1—O10	172.16 (5)	C21—N21—Zn1	106.38 (10)
O20—Zn1—O12	87.38 (5)	C22—N21—Zn1	104.13 (9)
O20—Zn1—N21	82.33 (5)	C22—N21—C21	112.43 (13)
O22—Zn1—N11	101.44 (5)	C24—N21—Zn1	109.17 (10)
O22—Zn1—O10	89.59 (5)	C24—N21—C21	112.87 (12)
O22—Zn1—O12	171.39 (5)	C24—N21—C22	111.32 (13)
O22—Zn1—N21	82.45 (5)	H21a—C21—N21	108.75 (8)
O22—Zn1—O20	84.05 (5)	H21b—C21—N21	108.75 (9)
C11—N11—Zn1	105.28 (10)	H21b—C21—H21a	107.6
C12—N11—Zn1	103.38 (9)	C20—C21—N21	113.99 (13)
C12—N11—C11	108.20 (13)	C20—C21—H21a	108.75 (9)
C14—N11—Zn1	118.91 (10)	C20—C21—H21b	108.75 (9)
C14—N11—C11	111.14 (12)	O20—C20—C21	119.63 (14)
C14—N11—C12	109.26 (12)	O21—C20—C21	116.24 (14)
H11a—C11—N11	109.33 (9)	O21—C20—O20	124.12 (15)
H11b—C11—N11	109.33 (8)	C20—O20—Zn1	115.23 (10)
H11b—C11—H11a	108.0	H22a—C22—N21	109.43 (8)
C10—C11—N11	111.50 (13)	H22b—C22—N21	109.43 (9)
C10—C11—H11a	109.33 (9)	H22b—C22—H22a	108.0
C10—C11—H11b	109.33 (9)	C23—C22—N21	111.03 (14)
O10—C10—C11	118.61 (14)	C23—C22—H22a	109.43 (9)
O11—C10—C11	116.27 (14)	C23—C22—H22b	109.43 (9)
O11—C10—O10	125.09 (15)	H23a—C23—C22	109.65 (9)
C10—O10—Zn1	113.59 (10)	H23b—C23—C22	109.65 (9)
H12a—C12—N11	109.39 (8)	H23b—C23—H23a	108.2
H12b—C12—N11	109.39 (8)	O22—C23—C22	110.07 (14)
H12b—C12—H12a	108.0	O22—C23—H23a	109.65 (9)
C13—C12—N11	111.19 (14)	O22—C23—H23b	109.65 (8)
C13—C12—H12a	109.39 (9)	C23—O22—Zn1	109.08 (10)
C13—C12—H12b	109.39 (9)	H22—O22—Zn1	122.3 (19)
H13a—C13—C12	110.40 (9)	H22—O22—C23	111.4 (19)
H13b—C13—C12	110.40 (9)	H24a—C24—N21	108.26 (8)
H13b—C13—H13a	108.6	H24b—C24—N21	108.26 (8)
O12—C13—C12	106.64 (14)	H24b—C24—H24a	107.4
O12—C13—H13a	110.40 (9)	C25—C24—N21	116.09 (14)
O12—C13—H13b	110.40 (9)	C25—C24—H24a	108.26 (9)
C13—O12—Zn1	109.53 (10)	C25—C24—H24b	108.26 (9)
H12—O12—Zn1	117.8 (18)	H25a—C25—C24	110.55 (9)
H12—O12—C13	108.9 (18)	H25b—C25—C24	110.55 (9)
H14a—C14—N11	108.83 (8)	H25b—C25—H25a	108.7
H14b—C14—N11	108.83 (8)	O23—C25—C24	105.92 (14)
H14b—C14—H14a	107.7	O23—C25—H25a	110.55 (9)
C15—C14—N11	113.65 (14)	O23—C25—H25b	110.55 (9)
C15—C14—H14a	108.83 (10)	H23—O23—C25	109.5
C15—C14—H14b	108.83 (9)	H2w—O1w—H1w	104 (2)
			
Zn1—N11—C11—C10	33.94 (10)	C11—N11—C12—C13	157.32 (13)
Zn1—N11—C12—C13	46.00 (10)	C11—N11—C14—C15	−67.40 (14)
Zn1—N11—C14—C15	55.04 (12)	C10—C11—N11—C12	−76.08 (15)
Zn1—O10—C10—C11	−10.61 (12)	C10—C11—N11—C14	163.96 (14)
Zn1—O10—C10—O11	167.43 (11)	C12—N11—C14—C15	173.27 (13)
Zn1—O12—C13—C12	39.80 (10)	C13—C12—N11—C14	−81.55 (15)
Zn1—N21—C21—C20	−15.46 (10)	N21—C21—C20—O20	9.05 (16)
Zn1—N21—C22—C23	45.13 (10)	N21—C21—C20—O21	−172.14 (14)
Zn1—N21—C24—C25	174.74 (10)	N21—C22—C23—O22	−54.90 (14)
Zn1—O20—C20—C21	3.57 (12)	N21—C24—C25—O23	−178.06 (15)
Zn1—O20—C20—O21	−175.14 (11)	C21—N21—C22—C23	−69.61 (14)
Zn1—O22—C23—C22	33.29 (10)	C21—N21—C24—C25	−67.19 (15)
N11—C11—C10—O10	−17.18 (16)	C20—C21—N21—C22	97.90 (15)
N11—C11—C10—O11	164.61 (14)	C20—C21—N21—C24	−135.15 (14)
N11—C12—C13—O12	−59.54 (14)	C22—N21—C24—C25	60.34 (14)
N11—C14—C15—O13	−172.88 (15)	C23—C22—N21—C24	162.63 (14)

### Bis{2-[bis(2-hydroxyethyl)amino]acetato}zinc(II) monohydrate Hydrogen-bond geometry (Å, º)

**Table:**

*D*—H···*A*	*D*—H	H···*A*	*D*···*A*	*D*—H···*A*
O12—H12···O21^i^	0.77 (2)	1.89 (2)	2.6558 (17)	176 (2)
O13—H13···O1*w*	0.79 (2)	1.89 (2)	2.6658 (19)	167 (1)
O22—H22···O11^ii^	0.78 (2)	1.82 (2)	2.5944 (17)	174 (3)
O23—H23···O21^iii^	0.73 (2)	1.99 (2)	2.7166 (17)	171 (3)
O1*w*—H1*w*···O13^iv^	0.79 (2)	1.95 (2)	2.739 (2)	177 (2)
O1*w*—H2*w*···O11^v^	0.79 (2)	1.97 (2)	2.7577 (18)	174 (2)
C12—H12*a*···O1*w*^vi^	0.94 (1)	2.65 (1)	3.563 (2)	163 (1)
C14—H14*b*···O13^i^	0.95 (1)	2.63 (1)	3.366 (2)	135 (1)
C15—H15*a*···O22	0.96 (1)	2.61 (1)	3.330 (2)	133 (1)
C15—H15*b*···O1*w*^vii^	0.96 (1)	2.61 (1)	3.564 (2)	172 (1)
C21—H21*a*···O23^viii^	0.94 (1)	2.52 (1)	3.457 (2)	177 (1)
C24—H24*b*···O10	0.95 (1)	2.53 (1)	3.171 (2)	125 (1)
C25—H25*a*···O23^ii^	0.98 (1)	2.62 (1)	3.589 (2)	170 (1)

Symmetry codes: (i) *x*, −*y*+3/2, *z*+1/2; (ii) *x*, −*y*+1/2, *z*−1/2; (iii) −*x*+1, *y*−1/2, −*z*+1/2; (iv) *x*, −*y*+3/2, *z*−1/2; (v) −*x*, *y*+1/2, −*z*−1/2; (vi) *x*, *y*, *z*+1; (vii) −*x*, −*y*+1, −*z*−1; (viii) −*x*+1, −*y*+1, −*z*+1.
